# Cannabidiol Effect on Cue-Induced Craving for Individuals with Opioid Use Disorder Treated with Buprenorphine: A Small Proof-of-Concept Open-Label Study

**DOI:** 10.1089/imr.2022.0070

**Published:** 2022-08-26

**Authors:** Joji Suzuki, Bianca Martin, Sara Prostko, Peter R. Chai, Roger D. Weiss

**Affiliations:** ^1^ Department of Psychiatry, Brigham and Women's Hospital, Boston, MA, USA.; ^2^ Harvard Medical School, Boston, MA, USA.; ^3^ Department of Emergency Medicine, Brigham and Women's Hospital, Boston, MA, USA.; ^4^ Department of Psychosocial Oncology and Palliative Care, Dana Farber Cancer Institute, Boston, MA, USA.; ^5^ The Koch Institute for Integrated Cancer Research, Massachusetts Institute of Technology, Cambridge, MA, USA.; ^6^ The Fenway Institute, Boston, MA, USA.; ^7^ McLean Hospital, Belmont, MA, USA.

**Keywords:** opioid use disorder, cannabidiol, cue-induced craving, buprenorphine

## Abstract

**Background:**

Opioid use disorder (OUD) remains a major public health concern. Despite the use of medications for OUD such as buprenorphine, the current gold-standard treatment, relapse in the context of increased craving remains common. Cannabidiol (CBD) has been shown to reduce cue-induced craving in individuals with OUD, but among those who were not receiving any buprenorphine treatment. This small proof-of-concept open-label study sought to evaluate the effect of CBD on cue-induced craving among individuals with OUD who were being actively treated with buprenorphine.

**Methods:**

Participants (*n* = 5) received CBD (Epidiolex^®^) 600 mg once daily for 3 consecutive days in an open-label manner. Primary outcome was cue-induced craving measured on a visual analog scale of 0 to 10, calculated as the difference in craving in response to drug-related versus neutral cues. The cue-reactivity paradigm was performed at baseline before CBD administration, and was repeated after 3 days of CBD. Secondary outcomes included scores on depression, anxiety, pain, opioid withdrawal, and side effects.

**Results:**

All participants were actively taking buprenorphine for an average of 37.8 months (range 1–120 months). Cue-induced craving was significantly lower after CBD dosing compared with baseline (0.4 vs. 3.2, paired *t*-test, *p* = 0.0046). No significant changes in scores for depression, anxiety, pain, or opioid withdrawal were noted. CBD was well tolerated, although one participant experienced moderate sedation; otherwise, no other adverse effects were reported.

**Conclusions:**

Given the high risk for bias in a small uncontrolled open label study such as this, results must be interpreted with caution. A larger adequately powered trial with a suitable control group is needed to confirm the finding that CBD may help to reduce cue-induced craving among individuals with OUD currently on buprenorphine treatment. Research should further evaluate whether adjunctive use of CBD can improve clinical outcomes for individuals with OUD maintained on buprenorphine. ClinicalTrials.gov (NCT04192370).

## Introduction

The United States is experiencing an unprecedented epidemic of opioid overdoses, currently fueled by the dramatic increase in fentanyl-related overdoses among individuals with opioid use disorder (OUD).^1^ Between April 2020 and April 2021, there were >100,000 overdose deaths, the highest number ever recorded in a 12-month period.^2^ Medications for OUD (MOUD), such as buprenorphine or methadone, can help to address this crisis because they help suppress illicit opioid use, increase retention in treatment, and reduce overdose mortality by up to 70%.^3–6^ Indeed, buprenorphine treatment is the first-line gold-standard treatment for OUD.^7^

Unfortunately, most patients relapse and discontinue MOUD treatment prematurely, due in part to the emergence of craving in response to exposure to drug-related cues and stressors.^8–10^ There is a lack of empirically supported pharmacologic adjuncts to standard MOUD that might help to reduce the relapse risk. Psychosocial interventions have robust empirical support for a variety of substance use disorders, but they have not been as helpful as hoped for in individuals with OUD receiving buprenorphine.^11^ Therefore, innovative interventions were urgently needed that help prevent individuals on buprenorphine from relapsing.

Cannabidiol (CBD), a nonaddictive and nonpsychoactive constituent of cannabis, has emerged as a potential treatment option for a variety of psychiatric and substance use disorders.^12–14^ The impact of cannabis itself on MOUD treatment outcomes remains unclear, with some showing improved outcomes, whereas others showing worse outcomes.^15,16^ CBD has low affinity for the CB1 and CB2 receptors, but instead demonstrates negative allosteric properties at CB1 as well as a broad range of pharmacologic actions through a variety of other receptors.^17,18^

In both animal and human models of addiction, CBD has been shown to reduce attentional bias to drug-related cues, cue-induced craving, and cue-induced drug reinstatement.^19–21^ This has led to studies evaluating the possible therapeutic applications of CBD for OUD. In prior studies of individuals with OUD, CBD reduced cue-induced craving for opioids for up to a week after administering CBD 400 or 800 mg for 3 days.^22,23^ However, those studies only enrolled individuals with OUD not receiving any MOUD including buprenorphine.

These results suggest that if applied to individuals with OUD receiving buprenorphine, CBD may potentially help attenuate the reactivity to triggers and reduce the risk of relapse. Accordingly, the aim of this small proof-of-concept open-label study was to evaluate the impact of CBD on cue-induced craving among individuals with OUD maintained on buprenorphine. It was hypothesized that individuals on buprenorphine will experience a reduction in cue-induced craving after receipt of CBD. Results will then inform whether a larger trial is warranted to further evaluate the effect of CBD in this patient population.

## Materials and Methods

### Overview

The study was a single-arm open-label pilot study of CBD 600 mg once daily for 3 days to assess CBD's impact on cue-reactivity in a within-subject design. The study was approved by the Mass General Brigham Human Research Committee.

### Setting

The study was conducted in the Clinical Trials Hub at Brigham and Women's Hospital, an urban academic medical center in Boston, MA. Recruitment and data collection occurred between September 2020 and December 2021.

### Study participants

Potential participants were recruited from local clinical programs that offer buprenorphine treatment, as well as from using online advertisements. Inclusion criteria were adults 18 years or older, having a DSM-5 diagnosis of OUD, and receiving treatment with buprenorphine. Exclusion criteria were the need for any inpatient level treatment for substance use or psychiatric disorders, history of any psychotic disorder, currently pregnant, hepatic enzymes greater than three times the upper normal limit, hypersensitivity to cannabinoids or sesame oil, and currently taking any medications with known significant pharmacokinetic interactions with CBD.

Participants were instructed to refrain from using any cannabis or cannabinoid products during the study. Owing to the impact of COVID-19 restrictions on participant recruitment during the study period, a decision was made to modify the protocol to allow inclusion of individuals receiving methadone as well. However, the study was completed by enrolling only those participants receiving buprenorphine.

### Overall study procedures

Interested individuals were initially screened for preliminary eligibility, after which eligible individuals were scheduled for their first study visit. At this visit, written informed consent was obtained, and baseline assessments and laboratory tests were conducted. Those who met full inclusion and exclusion criteria were then scheduled for two additional study visits, 3 days apart (Monday/Thursday or Tuesday/Friday). During the second visit (pre-CBD), participants completed the baseline cue-reactivity paradigm, after which they received their first dose of CBD 600 mg.

Participants were given two additional doses of CBD to take at home to take each day. At the third study visit (post-CBD), assessments and the cue-reactivity paradigm were repeated. Participants also completed a drug diary at home to report their intake of CBD, and reported any side effects experienced during the study.

### Cannabidiol

CBD (Epidiolex^®^) was purchased by the pharmacy as a 100 mg/mL oral solution, and drawn into oral syringes. Before study initiation, an investigational new drug exemption from the United States Food and Drug Administration was obtained for administration of CBD for this study. The first dose was administered through directly observed therapy to the participant, whereas the second and third doses were self-administered by the participant at home. The dose of 600 mg was selected to remain consistent with prior trials targeting psychiatric and substance use disorders where doses have typically ranged from 400 to 800 mg.^13,14^

### Craving assessments

At the beginning of both pre-CBD and post-CBD visits, participants were asked to report their craving for opioids (precue) before the cue-reactivity paradigm on a visual analog scale of 0 to 10. Participants then completed the cue-induced craving assessments. Participants were shown a total of 50 images, consisting of drug-related (40 total) and neutral (10 total) images on a computer screen using a standardized protocol used in previous studies.^24^ The order in which the cues were presented was randomized and counterbalanced. To limit habituation, drug-related images were not repeated between the pre-CBD and post-CBD cue-reactivity sessions.

Drug images were similarly matched to the neutral images in composition and style, and utilized images that have evoked strong responses in prior studies.^25^ After the stimuli presentation, participants rated their craving (postcue, neutral cue) on a visual analog scale of 0 to 10. The cue exposure procedure ended with a standardized relaxation and debriefing exercise.

### Assessments

The following assessments were completed at the baseline and post-CBD visits: Patient Health Questionnaire (PHQ-9),^26^ Generalized Anxiety Disorder-7 (GAD-7),^27^ Brief Pain Inventory (BPI),^28^ Positive and Negative Affect Scale (PANAS)^29^, and Clinical Opioid Withdrawal Scale (COWS).^30^

### Analytic strategy

Descriptive statistics were used to summarize the data. Cue-induced craving was calculated as the difference in postcue craving scores in response to drug-related cues minus craving scores in response to neutral cues. Cue-induced craving and all assessments were compared between baseline/pre-CBD and post-CBD visits using paired *t*-test, with alpha set at 0.05.

## Results

Participant characteristics are summarized in Table 1. Overall, they were mostly male (80%), averaged 37.8 years old (standard deviation [SD] 7.8), and mostly of nonwhite ethnicity (60%). Most had psychiatric comorbidities (major depression 80%, PTSD 60%, attention deficit hyperactivity disorder 60%, GAD 40%), as well as other substance use disorders (tobacco 80%, cocaine 20%, cannabis 20%, alcohol 20%). All participants were receiving buprenorphine treatment at the time of study enrollment, with a median duration of 20.0 months (range 1–120).

**Table 1.** Summary of Participant Characteristics

	Demographic variables
Age (SD)	37.8 Years (SD 7.8)
Sex, *n* (%)	*M*: 4 (80)
Race, *n* (%)	White: 2 (40.0)
	Black: 1 (20.0)
	Asian: 1 (20.0)
	Other: 1 (20.0)
Ethnicity	Hispanic: 1 (20)
	Non-Hispanic: 4 (80)
Lifetime psychiatric history, *n* (%)	
Major depression	4 (80.0)
Bipolar disorder	0
ADHD	3 (60.0)
GAD	3 (60.0)
Panic disorder	2 (40.0)
PTSD	3 (60.0)
Lifetime SUD history, *n* (%)	
OUD	5 (100.0)
Tobacco	4 (80.0)
Cocaine	1 (20.0)
Stimulant	0
Cannabis	1 (20.0)
Alcohol	1 (20.0)
Sedative/hypnotic	0
Median time on buprenorphine treatment, months (range)	20.0 (1–120)

ADHD, attention deficit hyperactivity disorder; GAD, generalized anxiety disorder; OUD, opioid use disorder; PTSD, post-traumatic stress disorder; SD, standard deviation; SUD, substance use disorder.

All participants self-reported ingesting all home doses of CBD dispensed in the study. One participant reported moderate sedation after the initial dose of CBD that persisted for up to 2 days until after all three doses were taken, but did not require medical intervention. No other adverse effects were noted among the participants.

Results of the assessments at baseline and after CBD are summarized in Table 2. Participants reported increased craving in response to exposure to drug-related stimuli at baseline as compared with neutral stimuli. The primary outcome, cue-induced craving, significantly decreased after receipt of CBD from 3.2 (SD 0.8) to 0.4 (SD 0.5) (paired *t*-test, *p* = 0.0046).

**Table 2.** Summary of Outcomes Before and After Receipt of Cannabidiol 600 mg for 3 Days

	Pre-CBD (baseline)	Post-CBD	*p*
PHQ-9 (range 0–27)	13.4 (6.3)	11.0 (7.3)	0.20
GAD7 (range 0–21)	13.0 (7.2)	10.8 (6.4)	0.051
BPI (range 0–10)			
Severity score	1.9 (2.7)	2.3 (3.0)	0.46
Interference score	1.1 (1.7)	2.9 (4.2)	0.19
PANAS (range 0–50)			
Positive score	22.4 (6.9)	26.4 (2.2)	0.16
Negative score	25.6 (13.0)	23.2 (12.8)	0.11
COWS (range 0–48)	1.2 (0.8)	2.4 (2.9)	0.28
Craving (range 0–10)			
Precue	3.2 (2.4)	1.0 (1.2)	0.19
Postcue	4.6 (1.5)	1.4 (1.1)	0.016
Neutral cue	1.4 (1.3)	1.0 (0)	0.034
Cue-induced craving	3.2 (0.8)	0.4 (0.5)	0.0046

BPI, Brief Pain Inventory; CBD, cannabidiol; COWS, Clinical Opioid Withdrawal Scale; GAD-7, Generalized Anxiety Disorder-7; PANAS, Positive and Negative Affect Scale; PHQ, Patient Health Questionnaire.

Postcue craving (4.6 vs. 1.4, paired *t*-test, *p* = 0.016) and neutral cue craving (1.4 vs. 1.0, paired *t*-test, *p* = 0.034) were also significantly decreased after receipt of CBD. Precue craving (3.2 vs. 1.0, *p* = 0.19), PHQ-9 (13.4 vs. 11.0, *p* = 0.20), GAD-7 (13.0 vs. 10.8, *p* = 0.051), BPI severity (1.9 vs. 2.3, *p* = 0.46), BPI interference (1.1 vs. 2.9, *p* = 0.19), PANAS positive affect (22.4 vs. 26.4, *p* = 0.16), PANAS negative affect (25.6 vs. 23.2, *p* = 0.11), and COWS scores (1.2 vs. 2.4, *p* = 0.28) were not significantly different between baseline/pre-CBD and post-CBD visits.

## Discussion

This is the first study to the authors' knowledge that investigated the impact of CBD on cue-induced craving among individuals with OUD who were actively being treated with buprenorphine. Consistent with this hypothesis, results showed that CBD significantly reduced cue-induced craving. Although still very preliminary, the findings extend prior research that found CBD can reduce cue-reactivity among OUD patients who were not receiving MOUD.^22,23^ Although buprenorphine remains one of the gold-standard treatments in improving clinical outcomes in patients with OUD, there is much room for improvement in the level of successful outcomes.^9,31,32^

Thus, research evaluating adjunctive treatments that can be offered in addition to buprenorphine is critically important. Cue-induced craving plays an important role in heightening the risk of relapse, suggesting that interventions that attenuate cue-reactivity may thus hold promise in improving outcomes for individuals already maintained on buprenorphine.^33,34^ In a study of individuals with OUD who completed laboratory-based cue-reactivity assessments, the strength of the cue-induced craving was significantly associated with shortened time to subsequent opioid relapse.^35^ In line with existing studies, CBD was generally well tolerated in this sample.

However, one of the study participants did experience moderate sedation that persisted for several days but not require any medical intervention, and was deemed to more likely than not related to the CBD.^36^ Given the importance of establishing safety of a new pharmacotherapy adjunct, further studies are needed to assess CBD's safety when combined with buprenorphine. Taken together, the growing evidence base of CBD's role in modulating the brain's response to drug-related cues in individuals with OUD suggests that more research is warranted to evaluate whether CBD may be a suitable pharmacologic adjunct to buprenorphine in improving clinical outcomes.

Although not statistically significant, the scores on BPI for pain severity and pain interference (i.e., the degree to which pain interferes with daily activities), as well as on COWS for opioid withdrawal, increased from pre-CBD to post-CBD. Although there is support for the analgesic properties of CBD in preclinical studies, there are no rigorous trials in humans to evaluate the effects of CBD alone on pain.^37,38^ In addition, no previous human trials have evaluated the effects of CBD on opioid withdrawal.^23^ Given the small sample size, these may be spurious findings, and further studies are needed to evaluate whether CBD improves or worsens pain and opioid withdrawal.

Nevertheless, there are important limitations to this study. This was a small uncontrolled proof-of-concept open-label study with a high risk for bias, therefore, the results remain preliminary. A larger study that is adequately powered that includes a suitable control group is needed to confirm the study findings. Although standard procedures was followed for assessing cue-reactivity, report of craving remains subjective and is susceptible to bias especially in an open-label study. All participants were treated with sublingual buprenorphine, making it problematic to generalize to individuals maintained on other formulations of buprenorphine, methadone, or extended-release naltrexone.

Finally, even though participants were instructed to refrain from using any cannabis or cannabinoid products during the trial, individuals with a cannabis use disorder were not excluded, nor was confirmed with toxicology testing whether participants were using any cannabinoid products or any other substances before or during the trial.

## Conclusions

In summary, CBD is a pharmacologic intervention that lacks any misuse liability that may hold promise as an adjunctive treatment to buprenorphine to reduce the risk of relapse by attenuating cue-reactivity. CBD is an FDA-approved pharmacotherapy, is not a controlled substance, and a recent randomized trial showed CBD's efficacy in reducing cannabis use individuals with cannabis use disorder.^39–41^ There are also numerous ongoing clinical trials of CBD for alcohol use disorder as well as OUD, highlighting the growing scientific interest in evaluating CBD as a potential pharmacotherapy for a variety of substance use disorders.

However, given the small open-label nature of this study, caution is warranted in interpreting these findings until they can be replicated in a larger trial with adequate controls. Specifically, future studies should employ a suitable control group, and additional reward- and stress-related neurocognitive processes that are implicated in the risk of relapse should be measured.^21,42–44^ Additional research is needed to better evaluate the potential role of CBD in improving clinical outcomes of individuals with OUD treated with buprenorphine.

## Abbreviations Used

ADHD: attention deficit hyperactivity disorder
BPI: Brief Pain Inventory
CBD: cannabidiol
COWS: Clinical Opioid Withdrawal Scale
GAD-7: Generalized Anxiety Disorder-7
MOUD: medications for OUD
OUD: opioid use disorder
PANAS: Positive and Negative Affect Scale
PHQ-9: Patient Health Questionnaire-9
PTSD: post-traumatic stress disorder
SD: standard deviation
SUD: substance use disorder
